# Identification of resistant carboxylesterase alleles in *Culex pipiens* complex via PCR-RFLP

**DOI:** 10.1186/1756-3305-5-209

**Published:** 2012-09-24

**Authors:** Hanying Zhang, Fengxia Meng, Chuanling Qiao, Feng Cui

**Affiliations:** 1 State Key Laboratory of Integrated Management of Pest Insects and Rodents, Institute of Zoology, Chinese Academy of Sciences, Beijing, 100101, China; 2 Department of Vector Biology and Control, National Institute for Communicable Disease Control and Prevention, China CDC, Beijing, 102206, China

**Keywords:** Carboxylesterase, Insecticide resistance, Genotype differentiation, Resistance monitor

## Abstract

**Background:**

Carboxylesterase overproduction is a frequently observed resistance mechanism of insects to organophosphate insecticides. As a major transmitter of human diseases, mosquitoes in the *Culex pipiens* complex have evolved 13 carboxylesterase alleles (*Ester*) that confer organophosphate resistance. Six alleles, *Ester*^*B1*^, *Ester*^*2*^, *Ester*^*8*^, *Ester*^*9*^, *Ester*^*B10*^, and *Ester*^*11*^, have been observed in field populations in China, sometimes co-existing in one population. To differentiate the carboxylesterase alleles found in these field populations, PCR-RFLP was designed for use in resistance monitoring.

**Results:**

Based on the DNA sequences of resistant and nonresistant carboxylesterase alleles, *Ester B* alleles were first amplified with PCR-specific primers and then digested with the restriction enzyme *Dra*I. In this step, *Ester*^*2*^ and *Ester*^*11*^ were differentiated from the other *Ester* alleles. When the other *Ester* B alleles were digested with the restriction enzyme *Xba*I, *Ester*^*B1*^ and the susceptible *C. p. pallens Ester* were screened out. *Ester*^*8*^ and *Ester*^*9*^ were differentiated from *Ester*^*B10*^ and the susceptible *C. p. quinquefasciatus* esterase allele, respectively, by amplifying and digesting the *Ester A* alleles with the restriction enzyme *Apa*LI. The effectiveness of the custom-designed PCR-RFLP was verified in two field mosquito populations.

**Conclusions:**

A PCR-RFLP based approach was developed to differentiate carboxylesterase alleles in *Culex pipiens* complex mosquitoes. These processes may be useful in monitoring the evolutionary dynamics of known carboxylesterase alleles as well as in the identification of new alleles in field populations.

## Background

Insecticides are vital to agricultural production and public health, particularly in countries with huge human populations, such as China. In China, large quantities of chemical insecticides have been used to control mosquitoes since the mid-1950s. Consequently, resistance has developed in vector mosquitoes, which makes their control increasingly difficult [1]. Mosquitoes in the *Culex pipiens* complex (Diptera: Culicidae) are common in temperate and tropical countries. These insects have been subjected to insecticide control in many places around the world [2]. Four subspecies comprise the complex: *C. p. quinquefasciatus*, *C. p. pallens*, *C. p. pipiens*, and *C. p. molestus*. *C. p. quinquefasciatus* and *C. p. pallens* are prevalent in South China and North China, respectively [3].

Global surveys of insecticide resistance have indicated that one of the major mechanisms of resistance is the increased detoxification in resistant individuals [4]. Three primary detoxifying enzymes involved in insecticide resistance are carboxylesterase (or esterases), glutathione-S-transferases, and P450 monooxygenases, which are qualitatively or quantitatively changed to confer resistance [5]. Esterase overproduction is a common resistance mechanism of *C. pipiens* complex mosquitoes to organophosphate (OP) insecticides. This process is achieved mainly by gene amplification or occasionally by gene up regulation [6,,^7^]. Some studies indicated that gene duplication or amplification may be a more common adaptive evolutionary mechanism in arthropods, and that certain genomic loci may be “hot spots” for gene duplication, as evidenced by parallel evolution in several arthropod species [8].

In the *C. pipiens* complex, two carboxylesterase loci, *Est-3* (encoding esterase A) and *Est-2* (encoding esterase B), are amplified in the genome and subsequently confer resistance to insecticides [9,,^10^]. *Est-3* and *Est-2* are usually in complete linkage disequilibrium when amplified and are thus referred to as the *Ester* superloci [11]. To date, 13 alleles that confer insecticide resistance have been identified at the *Ester* superloci in the *C. pipiens* complex. These alleles (with the corresponding overproduced esterases indicated in parentheses) are *Ester*^*A1*^ (A1), *Ester*^*2*^ (A2-B2), *Ester*^*4*^ (A4-B4), *Ester*^*5*^ (A5-B5), *Ester*^*8*^ (A8-B8), *Ester*^*9*^ (A9-B9), *Ester*^*B1*^ (B1), *Ester*^*B6*^ (B6), *Ester*^*B7*^ (B7), *Ester*^*B10*^ (B10), *Ester*^*11*^ (A11-B11), *Ester*^*B12*^ (B12), and *Ester*^*A13*^ (A13) [2,,7,,^12^-^15^]. Some resistant alleles are distributed globally. For instance, *Ester*^*2*^ is found in Africa, Asia, Europe, North America, and the Caribbean [12,,^16^]. Meanwhile, some resistant alleles are found in restricted geographic areas. For example, *Ester*^*8*^, *Ester*^*9*^, *Ester*^*B10*^, and *Ester*^*11*^ are endemic to China [14,,^17^-^19^]. Thus, an unusual diversity of *Ester* alleles is observed in field populations in China, where *Ester*^*B1*^, *Ester*^*2*^, *Ester*^*8*^, *Ester*^*9*^, *Ester*^*B10*^, and *Ester*^*11*^ have been reported to coexist in one population [13,,^14^]. The polymorphism of resistant esterase alleles may be the result of changes in insecticide use and/or of a recent contact between relatively isolated treated areas through migration [13].

The identification and monitoring of the frequencies of these resistant *Ester* alleles in field populations of mosquitoes are vital not only to the control of vector mosquitoes and the diseases they transmit, but also to field evolutionary studies of these alleles. Current methods of genotyping or allele identification include restriction fragment length polymorphism (RFLP) identification, random amplified polymorphic detection (RAPD), amplified fragment length polymorphism detection (AFLPD), polymerase chain reaction (PCR), DNA sequencing, allele specific oligonucleotide (ASO) probes, and hybridization to DNA microarrays or beads. The most applied method in studies of *Culex Ester* alleles is the starch gel electrophoresis, which only reveals the phenotype of *Ester* alleles instead of the genotype. In this paper, we designed specific primers and selected the appropriate restriction endonucleases for use in PCR-RFLP to differentiate between resistant and nonresistant *Ester* alleles in *C. pipiens* complex mosquitoes. The effectiveness of the PCR-RFLP system was verified in field-collected mosquitoes.

## Methods

### Mosquitoes

Eight standard *C. pipiens* complex strains were used. These strains are S-LAB, which is an OP-susceptible *C. p. quinquefasciatus* strain without increased esterase activity [20]; BJSU, an insecticide-susceptible *C. p. pallens* strain collected from Beijing in the 1970s and laboratory-reared for over 40 years without insecticide exposure; and SB1, SA2, MAO2, LING, KARA2, and WU, which are homozygous *C. p. quinquefasciatus* for *Ester*^*B1*^, *Ester*^*2*^, *Ester*^*8*^, *Ester*^*9*^, *Ester*^*B10*^, and *Ester*^*11*^, respectively [13,,18,,^21^]. Two field populations were also obtained: R-SG, which was *C. p. quinquefasciatus* and collected from Foshan in Guangdong Province (South China) in July 2007, and TAA, which was *C. p. pallens* and collected from Tai’an in Shandong Province (North China) in July 2010 [22].

### DNA isolation and PCR-RFLP

Genomic DNA from intact single adult mosquitoes or from their head-thoraces was extracted using the cetyl trimethyl ammonium bromide (CTAB) method, as described by Roger and Bendich [23]. Based on the GenBank *Ester* allele sequences (Tables 1 and 2), the *EsterB* allele fragments were amplified using the Bdir (5^′^AGCTTAACCGTTCAGACCAA3^′^) and Brev (5^′^CAGTCCAACGTTTCGGTCCA3^′^) primers, whereas the *EsterA* alleles were amplified using the Adir (5^′^CTTTATGAGAGGATCTAGTGG3^′^) and Arev (5^′^CAAGCTTCACGACACATCTC3^′^) primers. The 50 μl PCR mixture contained 1 μl of genomic DNA (50 ng/μl to 100 ng/μl), 0.25 μM of each primer, 0.2 mM of each dNTP, and 2.5 units of LA Taq polymerase (Takara, Otsu, Shiga, Japan) in a 1× reaction buffer. PCR was performed on a thermocycler (Mastercycler Gradient, Eppendorf, Hamburg, Germany) at a denaturing step of 94°C for 4 min, followed by 35 cycles of 94°C for 45 s, 58°C for 45 s, and 72°C for 2 min and 40 s for the *Ester B* gene or 1 min and 10 s for the *EsterA* gene. A final extension of 10 min at 72°C followed. After verification via 1% agarose gel electrophoresis, the PCR products were digested with the restriction enzymes *Dra*I or *Xba*I for *EsterB* and *Apa*LI for *EsterA* at 37°C for 2 h in accordance with the manufacturer’s instructions (BioLabs, Ipswich, MA, USA). The digested products were electrophoresed in 2% agarose gel and photographed under ultraviolet light illumination using a gel imaging system (Gene Company, Hong Kong, China). 

**Table 1:** PCR and RFLP products of esterase B alleles

**Alleles**	**GenBank ID**	**PCR (bp)**	***Dra*I digestion (bp)**		***Xba*I digestion (bp)**
			**Predicted**	**In gel**	
B1	M32328	945	512, 369, 64	512, 369	945
B2	Z86069	941	677, 244	677, 244	941
B8	EF174325	2443	951, 888, 424, 89, 69, 22	951, 424, 180	2443
B9	JQ866911	936	402, 292, 242	402, 292, 242	936
B11	EF174328	942, 1165	519, 423, 742, 423	742, 519, 423	942, 1165
B10	EF174326	2442	951, 888, 424, 89, 68, 22	951, 424, 180	2442
Sp-B^1^	JQ812615	942	519, 375, 48	519, 375	353, 589
Sq-B^2^	JQ341053	936	402, 292, 242	402, 292, 242	936

^1^Susceptible *Ester B* allele of *C. p. pallens.*

^2^Susceptible *Ester B* allele of *C. p. quinquefasciatus.*

**Table 2:** PCR and RFLP products of esterase A alleles

**Alleles**	**GenBank ID**	**PCR (bp)**	***Apa*LI digestion (bp)**
B1-A	JQ780068	714	321, 393
A2	Z86069	713	321, 392
A8	AJ302089	713	98, 223, 392
A9	AJ302090	713	98, 615
A11	EF174327	713	98, 223, 409
B10-A	AJ302090	713	98, 615
Sp-A^1^	JQ812614	713	321, 392
Sq-A^2^	JQ812613	713	321, 392

^1^Susceptible *Ester A* allele of *C. p. pallens.*

^2^Susceptible *Ester A* allele of *C. p. quinquefasciatus.*

### Starch gel electrophoresis

Single adult mosquitoes from the R-SG and TAA field populations were cut into two parts: the head-thorax and the abdomen. The abdomen of each mosquito was homogenized in 10 μl distilled water using a pestle. The homogenate was spread onto a Whatman Grade No. 3 filter paper (3 mm × 8 mm) and analyzed via starch gel electrophoresis to identify the carboxylesterase phenotypes [24]. The head-thorax was used in PCR-RFLP for carboxylesterase genotype identification. At least 10 individuals from each population were analyzed.

## Results and discussion

### Discrimination of carboxylesterase alleles in standard strains via PCR-RFLP

**Figure 1:**
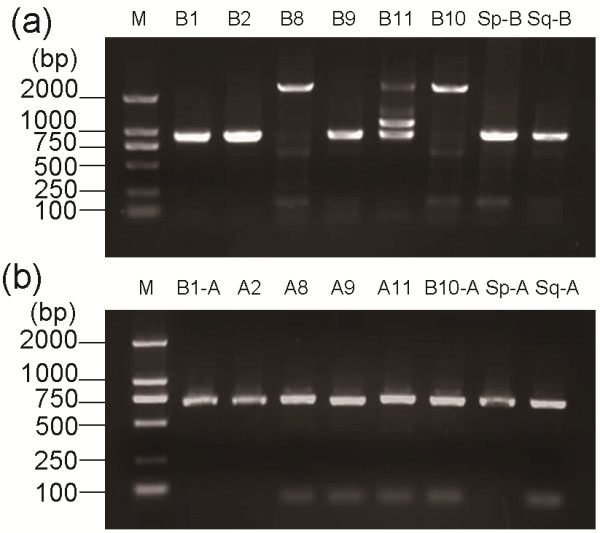
** Agarose gel electrophoresis for PCR products of (a) esterase B and (b) esterase A from standard *Culex pipiens* complex strains.** M, Marker D2000; B1 from the SB1 strain; A2-B2 from the SA2 strain; A8-B8 from the MAO2 strain; A9-B9 from the LING strain; A11-B11 from the WU strain; B10 from the KARA2 strain; Sp, susceptible esterases of *C. p. pallens* from the BJSU strain; Sq, susceptible esterases of *C. p. quinquefasciatus* from the S-LAB strain.

To discriminate the carboxylesterase alleles, the *Ester B* allele fragments were first amplified by PCR. Most PCR products of the *Ester B* alleles were approximately 900 bp long, whereas those of *Ester B8* and *Ester B10* were approximately 2400 bp in length (Figure. 1a and Table 1). Meanwhile, the PCR product of *Ester B11* showed two predominant bands that correspond to 942 and 1165 bp fragments. Sequencing data show that the 942 bp band is *Ester B11*, whereas the 1165 bp band had a 223 bp insertion in one *Ester B11* intron. This insertion may have originated from gene recombination or transposition in the genome. Whether this insertion affects the insecticide resistance in mosquitoes remains to be determined.

**Figure 2:**
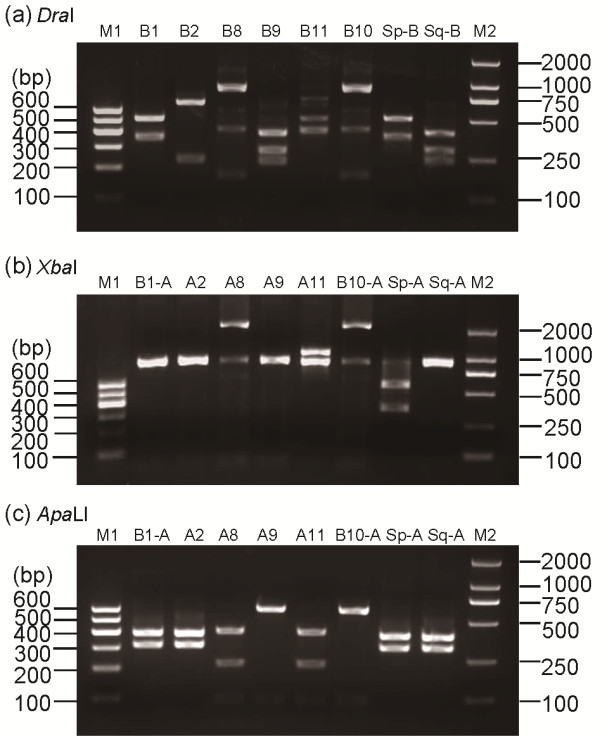
** (a)*Dra*I and (b)*Xba*I PCR**-**RFLP profiles of esterase B and (c)*Apa*LI PCR**-**RFLP profiles of esterase A from standard *C. pipiens* complex strains.** M1, Marker I. M2, Marker D2000. The standard strains are the same as those described in Figure 1.

After the PCR products were digested by the restriction enzyme *Dra*I, the *Ester B* alleles showed various digestion patterns. Given the resolution limit of the 2% agarose gel electrophoresis, some *Ester B* allele digestion patterns in the agarose gel electrophoresis differed from the predicted theoretical patterns (Table 1). In the electrophoresis graph (Figure. 2a), the *Ester B2* (lane 2) and *Ester B11* (lane 5) patterns were unique, thereby allowing the differentiation of *Ester*^*2*^ and *Ester*^*11*^ from the other alleles. The 223 bp insertion in the 1165 bp *Ester B11* PCR product resulted in the appearance of a 742 bp excess band during digestion. Based on *Dra*I digestion, three pairs of *Ester B* alleles were distinguished by differences in their migration patterns (Figure. 2a): *Ester B1* (lane 1) and the susceptible *C. p. pallens Ester B* (lane 7); *Ester B8* (lane 3) and *Ester B10* (lane 6); *Ester B9* (lane 4) and the susceptible *C. p. quinquefasciatus Ester B* (lane 8).

The PCR products of the three pairs of *Ester B* alleles were digested by the restriction enzyme *Xba*I (Figure. 2b) to allow differentiation among members of the same pair. The pair consisting of *Ester B1* (lane 1) and the susceptible *C. p. pallens Ester B* (lane 7) displayed different digestion profiles, which allowed the differentiation of *Ester*^*B1*^ from the susceptible *C. p. pallens*. However, the *XbaI* enzyme could not discriminate *Ester B8* (lane 3) from *Ester B10* (lane 6), or *Ester B9* (lane 4) from the susceptible *C. p. quinquefasciatus Ester B* (lane 8). At this step, four *Ester* alleles had already been distinguished from one another: *Ester*^*2*^, *Ester*^*11*^, *Ester*^*B1*^, and the susceptible *C. p. pallens*.

**Figure 3:**
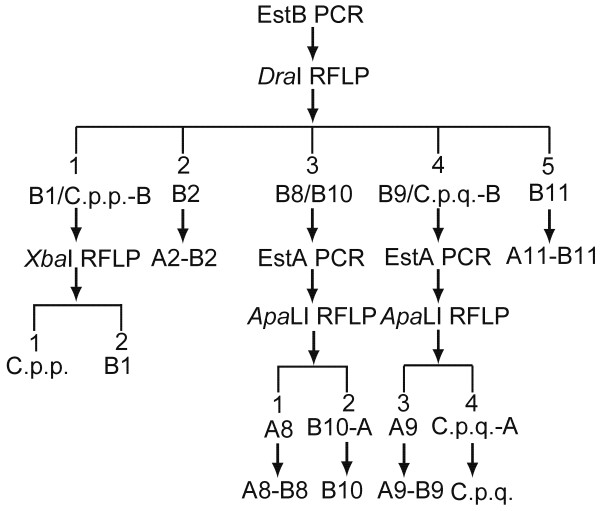
** Diagrammatic summary of PCR**-**RFLP discrimination of esterase alleles from *C. pipiens* complex mosquitoes.**

*Ester*^*8*^ and *Ester*^*9*^ were differentiated from *Ester*^*B10*^ and the susceptible *C. p. quinquefasciatus*, respectively, by amplifying an approximately 700 bp fragment of their *Ester A* via PCR using specific primers (Figure. 1b and Table 2) and then digesting the fragment with the restriction enzyme *Apa*LI. *Ester A8* (lane 3) displayed digestion profiles that differ from that of the *Ester*^*B10*^*Ester A* (lane 6) (Figure. 2c). In addition, *Ester A9* (lane 4) displayed profiles that differ from those of the susceptible *C. p. quinquefasciatus Ester A* (lane 8) (Figure. 2c). Thus, the six resistant *Ester* alleles *Ester*^*B1*^, *Ester*^*2*^, *Ester*^*8*^, *Ester*^*9*^, *Ester*^*B10*^, and *Ester*^*11*^, as well as the susceptible alleles, were correctly identified (Figure. 3).

*Ester*^*A1*^, *Ester*^*4*^, *Ester*^*5*^, *Ester*^*B6*^, *Ester*^*B7*^, *Ester*^*B12*^, and *Ester*^*A13*^ are found in other places in the world. However, given that no sequence information on the association of *Ester*^*B6*^, *Ester*^*B7*^, and *Ester B* to *Ester*^*A1*^ and *Ester*^*A13*^ is available, and that only a partial sequence of *Ester*^*B12*^ is currently known, the designed PCR-RFLP cannot be used to differentiate these alleles. The predicted PCR product sizes for *Ester B4* and *Ester B5* are 1289 and 1337 bp according to the *Ester*^*4*^ and *Ester*^*5*^ sequences, respectively; these values deviate from those of other *Ester B* alleles (Table 1). However, the *Dra*I digestion profiles of *Ester B4* and *Ester B5* (764 and 467 bp for *Ester B4*, and 770 and 518 bp for *Ester*^*5*^) may be difficult to distinguish in 2% agarose gel electrophoresis. No *Xba*I cut site exists in *Ester B4* and *Ester B5*. The predicted PCR products of *Ester A4* and *Ester A5* are 714 and 713 bp, respectively. Only *Ester A5* can be cut by *Apa*LI to produce 321 and 392 bp bands. Thus, the designed PCR-RFLP can identify eight resistant *Ester* alleles (*Ester*^*B1*^, *Ester*^*2*^, *Ester*^*4*^, *Ester*^*5*^, *Ester*^*8*^, *Ester*^*9*^, *Ester*^*B10*^, and *Ester*^*11*^) and two susceptible alleles.

### Identification of carboxylesterase alleles in mosquitoes from field populations

**Figure 4:**
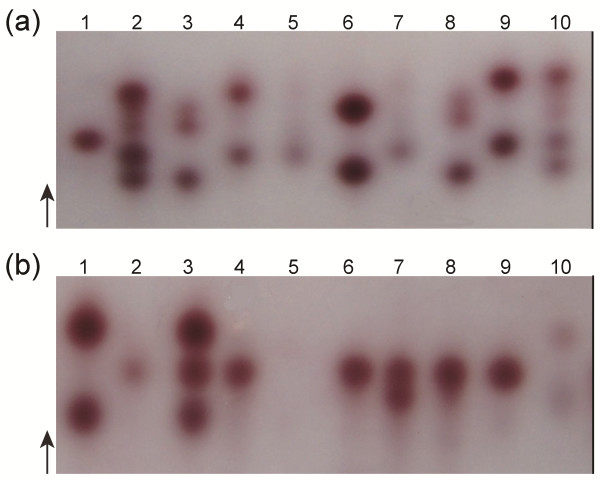
** High-activity esterases of single adults from the R**-**SG and TAA field-collected populations, as determined via starch gel electrophoresis.** (**a**) R-SG population: 1, B1 from the SB1 strain as control; 2–5 and 7–10, R-SG field samples; 6, A2-B2 from the SA2 strain as control. (**b**) TAA population: 1, A2-B2 from the SA2 strain as the control; 2–9, TAA field samples; 10, A11-B11 from the WU strain as control.

**Figure 5:**
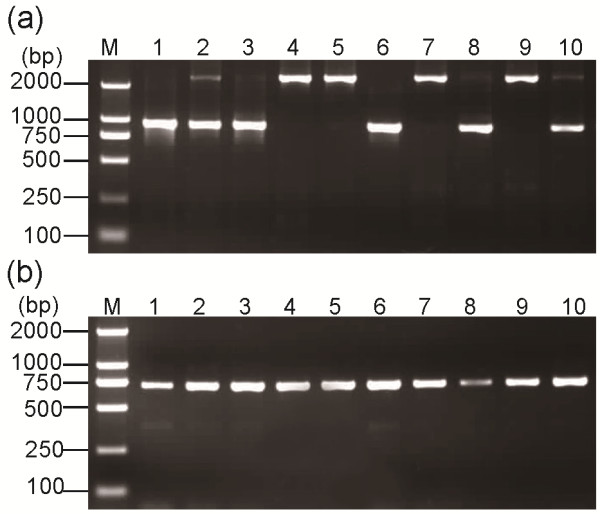
**Agarose gel electrophoresis of the PCR products of (a) esterase B and (b) esterase A from the R**-**SG population.** M, Marker D2000; 1, B1 from the SB1 strain as control; 6, A2-B2 from the SA2 strain as control; 2–5 and 7–10, R-SG field samples

**Figure 6:**
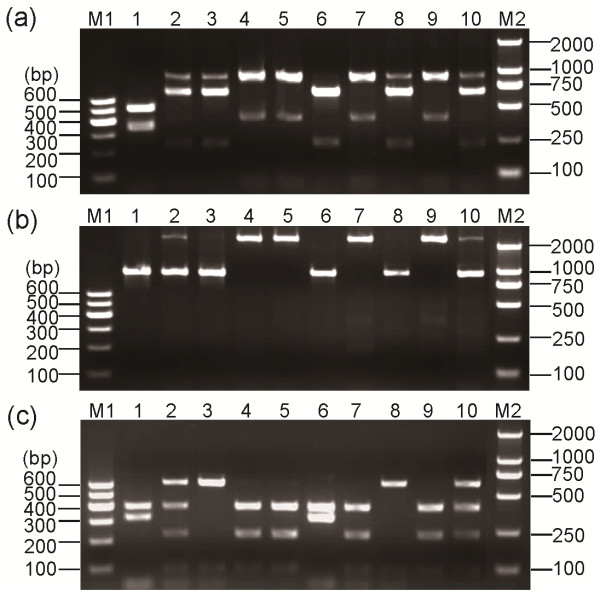
**(a)*Dra*I and (b)*Xba*I PCR**-**RFLP profiles of esterase B and (c)*Apa*LI PCR**-**RFLP profiles of esterase A from the R-SG population.** M1, Marker I; M2, Marker D2000; 1–10, as described in Figure 5

The phenotype and genotype of carboxylesterase alleles in mosquitoes from two field populations, R-SG (*C. p. quinquefasciatus*) and TAA (*C. p. pallens*), were identified through starch gel electrophoresis and PCR-RFLP. Of the eight mosquitoes from the R-SG population, Nos. 4, 5, 7, and 9 showed the A8-B8 phenotype in starch gel electrophoresis (Figure. 4a). PCR-RFLP results confirm that these mosquitoes were homozygous with *Ester*^*8*^ (Figure 5 and Figure 6). Starch gel electrophoresis results suggest that the Nos. 3 and 8 mosquitoes are heterozygous to A2-B2 and A9-B9 or A9-B10 and A9-B9 because of the similar mobilities of A2-B2 and A9-B10 [14]. However, PCR-RFLP results indicate that these mosquitoes are actually heterozygous to A9-B10 and B2 (Figure 5 and Figure 6). Three esterase Bs and two esterase As were found in the starch gel electrophoresis profile of Nos. 2 and 10 mosquitoes (Figure. 4a). The whole set of PCR-RFLP shows that the No. 2 mosquito expressed A8-B8, A9-B9, and B2, whereas the No. 10 mosquito expressed A8-B8, A9-B10, and B2 (Figure 5 and Figure 6). The fewer than expected *Dra*I digestion bands of esterase B alleles in mosquito No. 2 (Figure 6A, lane 2) could be due to the higher gene copy number of B2 in the genome over B8 and B9. Without the PCR-RFLP results, the differentiation of the phenotypes of the two mosquito types would have been difficult. 

**Figure 7:**
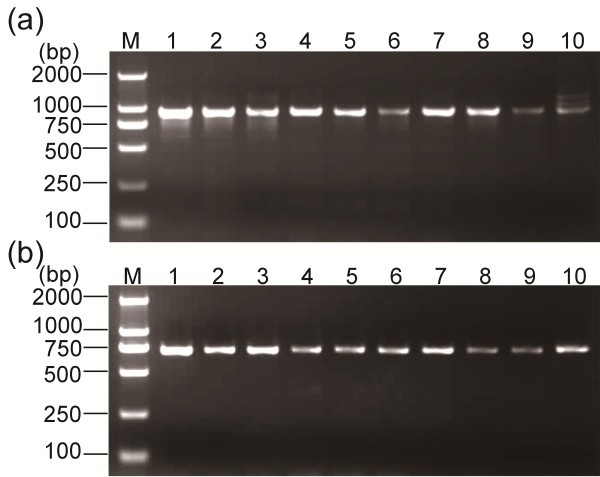
**Agarose gel electrophoresis of the PCR products of (a) esterase B and (b) esterase A from the TAA population.** M, Marker D2000; 1, A2-B2 from the SA2 strain as control; 2–9, TAA field samples; 10, A11-B11 from the WU strain as control

**Figure 8:**
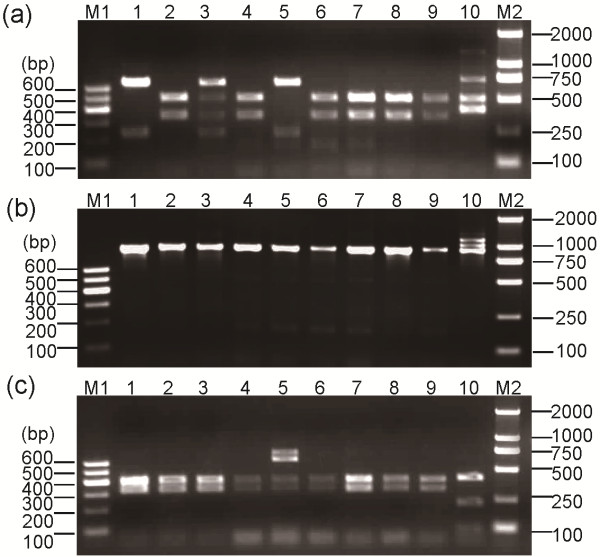
** (a)*Dra*I and (b)*Xba*I PCR**-**RFLP profiles of esterase B and (c)*Apa*LI PCR**-**RFLP profiles of esterase A from the TAA population.** M1, Marker I; M2, Marker D2000; 1–10, as described in Figure 7.

Most of the eight mosquito types from the TAA population (Figure. 4b) overproduced esterase B1 (No. 2, 4, 6, 7, 8, and 9 mosquitoes). The No. 3 mosquito was heterozygous to B1 and A2-B2. These phenotypes were confirmed by PCR-RFLP (Figure 7 and Figure 8). The No. 5 individual did not appear to have overproduced esterases based on the light-colored band in the starch gel electrophoresis. However, PCR-RFLP results indicate the presence of A2-B2 and A9 (Figure 7 and Figure 8). The results for the two field populations show that the A2-B2 and A9-B9 linkages are not always established. This result deviates from the usual observations and theories [7]. By now the recombination event leading to the existence of three esterase B alleles in one individual mosquito has only been observed in field populations of China [13 and this study]. The dislinkage between A2 and B2 or A9 and B9 probably results from the rare recombination event among these esterase alleles. 

## Conclusions

The custom-designed PCR-RFLP method can be used to differentiate the insecticide-resistant esterase alleles existing in China. The method can help monitor the evolutionary dynamics of these esterase alleles and identify new esterase alleles in field populations of *C. pipiens* complex.

## Competing interests

The authors declare that they have no competing interests.

## Authors' contributions

HZ performed the study, analyzed the data and drafted the manuscript. FM supplied the BJSU strain and helped draft the manuscript. CQ supervised the study and helped draft the manuscript. FC conceived, designed the study, and finalized the manuscript. All authors read and approved the final version of the manuscript.
